# Nocturnal Light Pollution Induces Weight Gain in Mice and Reshapes the Structure, Functions, and Interactions of Their Colonic Microbiota

**DOI:** 10.3390/ijms23031673

**Published:** 2022-01-31

**Authors:** José Sarmiento, Rodrigo Pulgar, Dinka Mandakovic, Omar Porras, Carlos A. Flores, Diego Luco, Carlos A. Trujillo, Briam Díaz-Esquivel, Cinthya Alvarez, Alejandro Acevedo, Marcelo A. Catalán

**Affiliations:** 1 Instituto de Fisiología, Facultad de Medicina, Universidad Austral de Chile, Valdivia 5090000, Chile; jsarmien@uach.cl (J.S.); d.luco.p@gmail.com (D.L.); carlos.trujillo@uach.cl (C.A.T.); bryam.28.93@gmail.com (B.D.-E.); cinthya.alvarez1991@gmail.com (C.A.); 2 Escuela de Graduados, Facultad de Ciencias Veterinarias, Universidad Austral de Chile, Valdivia 5090000, Chile; 3 Instituto de Nutrición y Tecnología de los Alimentos (INTA), Universidad de Chile, Santiago 7810000, Chile; rpulgar@inta.uchile.cl (R.P.); omar.porras@inta.uchile.cl (O.P.); a.acevedo.aracena@gmail.com (A.A.); 4 GEMA Center for Genomics, Ecology and Environment, Universidad Mayor, Santiago 8580745, Chile; dinka_slavja@hotmail.com; 5 Centro de Estudios Científicos, Valdivia 5110466, Chile; cflores@cecs.cl

**Keywords:** light pollution, chronodisruption, body mass, microbiota

## Abstract

In mammals, the daily variation in the ecology of the intestinal microbiota is tightly coupled to the circadian rhythm of the host. On the other hand, a close correlation between increased body weight and light pollution at night has been reported in humans and animal models. However, the mechanisms underlying such weight gain in response to light contamination at night remain elusive. In the present study, we tested the hypothesis that dim light pollution at night alters the colonic microbiota of mice, which could correlate with weight gain in the animals. By developing an experimental protocol using a mouse model that mimics light contamination at night in urban residences (dLAN, dim light at night), we found that mice exposed to dLAN showed a significant weight gain compared with mice exposed to control standard light/dark (LD) photoperiod. To identify possible changes in the microbiota, we sampled two stages from the resting period of the circadian cycle of mice (ZT0 and ZT10) and evaluated them by high-throughput sequencing technology. Our results indicated that microbial diversity significantly differed between ZT0 and ZT10 in both LD and dLAN samples and that dLAN treatment impacted the taxonomic composition, functions, and interactions of mouse colonic microbiota. Together, these results show that bacterial taxa and microbial metabolic pathways might be involved with the mechanisms underlying weight gain in mice subjected to light contamination at night.

## 1. Introduction

Lifestyle in the modern era is associated with profound changes in the diet, where irregular feeding times and high consumption of processed foods are common behaviors [1]. Moreover, changes in the diet such as high caloric diet intake and sedentarism have been associated with higher rates of overweight and obesity [2,3]. Other non-dietary related features of the modern lifestyle are also related to overweight and/or obesity. For instance, exposure to artificial light at night is associated with body weight gain in humans [4] and animal models [5,6]. In this regard, it is not fully understood how exposure to light at night induces weight gain; however, rotating shift work, bright streetlights, the use of light-emitting devices at night, and social jet lag have been linked to many pathologies, including obesity, altered immune system, oxidative stress, and cancer (reviewed in [7]).

The risk factors mentioned above cause chronodisruption, a term used for disruption of the circadian cycle [8]. At the molecular level, rhythmic expression of genes, such as *Period1/2*, *Clock*, *Bmal1*, *Cry1*, and *Rev-erbα*, among others, constitute the circadian molecular oscillator that controls the function of different organs and systems at the cellular level. The combined effect of several *zeitgebers*, such as light and mealtime, results in the synchronization of the circadian clock, which comprises the central, suprachiasmatic nucleus (SCN), and diverse peripheral clocks [9]. Furthermore, food composition and feeding time modulate the clock of peripheral tissues such as the intestine, liver, and kidney [10].

Circadian regulation plays a crucial role in metabolic homeostasis [11,12]. In the case of weight gain caused by light exposure at night, it has been shown that photoperiod-induced chronodisruption severely impairs metabolic homeostasis by altering liver function [5]. Furthermore, other mechanisms by which chronodisruption can influence the development of altered metabolic homeostasis occur via changes in the intestinal microbiome [1]. In this regard, a recent large-scale study using human stool samples identified circadian rhythms in microbiota [13], where a very accurate prediction of diabetes type II disease could be inferred from intestinal microbiota analysis, which is characterized by arrhythmic taxa in diabetic patients. Hence, although the effect of chronodisruption on the intestinal microbiota functions has received less attention than the host, evidence exists of its potential impact on weight gain. From the functional point of view, some metabolites generated by microbial activity, such as short-chain fatty acids (SCFAs), act as a strong signal that entrains diverse peripheral clocks in the intestinal and liver tissues, among other peripheral organs [14]. Thus, the modulation of peripheric organs by the intestinal microbiota’s metabolic activity might be considered a potential target for regulating body mass. Indeed, colonic administration of propionate for six months effectively reduced weight and fat tissue in humans displaying overweight [15]. Similar findings have been reported in mice using acetate, propionate, butyrate, or a mixture of SCFAs to avoid weight gain and insulin resistance induced by a high-fat diet [16].

Significant advances in microbiota analysis have shown a correlation between intestinal microbiota and the type of diet or presence of metabolic diseases (i.e., diabetes type II) [17,18]. However, other conditions that lead to an accelerated weight gain, such as light contamination during the resting period, have not been addressed from this perspective. In this study, we tested the hypothesis that weight gain caused by the exposure of mice to dim light at night is associated with changes in their colonic microbiota. Our results show that dim light at night (dLAN) exposure alters the composition and functions associated with the mouse colonic microbiota and dramatically impairs the interaction network of bacterial communities.

## 2. Results

### 2.1. Dim Light at Night Exposure Increases Body Mass in Mice

**Figure 1:**
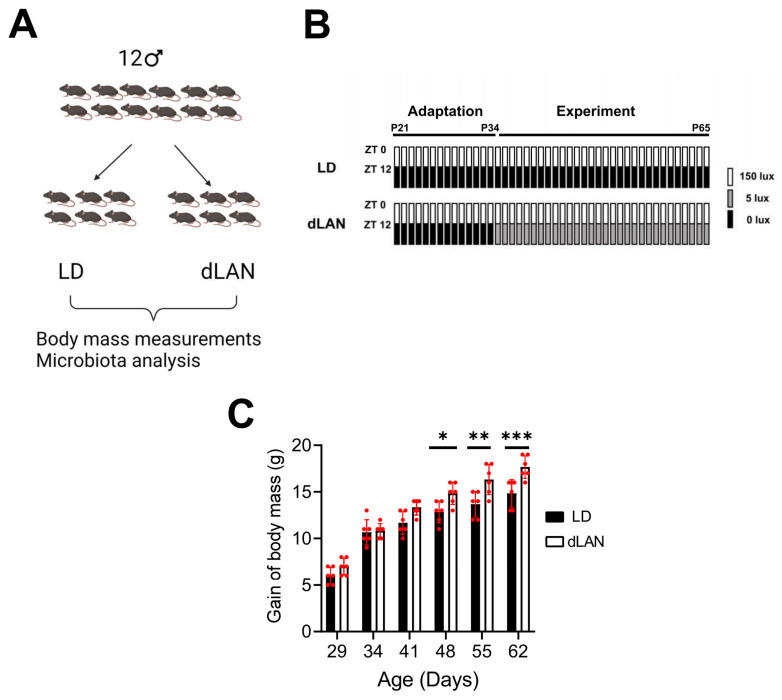
Control (LD) vs. dim light at night (dLAN) protocols. (**A**) Cartoon showing the experimental design. (**B**) All mice were subjected to the adaptation phase for fourteen days (until postnatal day (P34)) at LD followed by LD or dLAN protocol for thirty days (until P65). (**C**) Gain of body mass displayed by mice exposed to LD (black bars, *n* = 6) or dLAN (open bars, *n* = 6). *, **, and *** correspond to *p*-values less than 0.05, 0.01, and 0.001 obtained by two-way ANOVA, Sidak’s multiple comparison test (LD vs. dLAN). Bars and error bars (red color) correspond to the means ± SD, respectively.

Exposure to light at night has been associated with a gain in body weight in humans and animal models. To validate our homemade LED-based illumination system, we evaluated the effect of exposing mice to dim light at night (dLAN, Figure 1). The animal cohort used in the study and the scheme of the experimental protocol used are shown in Figure 1A,B. As seen in Figure 1C, we found that from the third week of treatment (day 48 in the *x*-axis), body mass gain of mice exposed to dLAN was significantly higher than that from control mice (LD, *p* < 0.05; two-way ANOVA, Sidak’s multiple comparison test).

### 2.2. Dim Light at Night Impacts the Taxonomic Composition of Mouse Colonic Bacterial Communities

**Figure 2:**
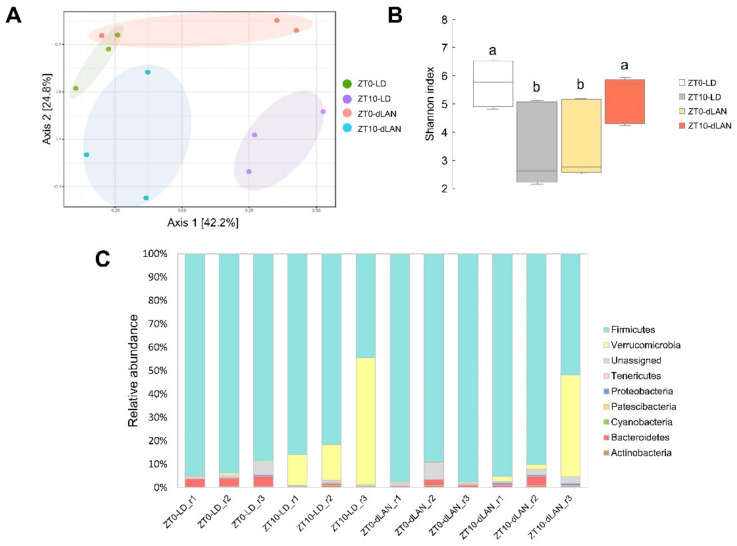
Diversity metrics and taxonomic structure of bacterial communities from LD and dLAN samples. (**A**) PCA ordination diagram of beta diversity of the microbial samples (Bray Curtis index; PERMDISP *p*-value > 0.05; PERMANOVA F value: 3.0966; R-squared: 0.5373; *p*-value < 0.001). (**B**) Shannon index. Horizontal bars within boxes represent median, where the top and bottom of each box represent the 75th and 25th quartiles of the bacterial communities. Bars with different letters indicate statistically significant differences (two-way ANOVA *p* < 0.05, Tukey’s multiple comparison test). (**C**) Bacterial phyla relative abundance in all samples.

Since we pursued the variations in colonic bacterial communities from LD- and dLAN-treated mice, we evaluated their fecal microbiomes considering two representative steps of the day: at the zeitgebers ZT0/24 and ZT10 (resting period) by high-throughput sequencing (HTS) technology. It is important to note that the feces used in the analysis were from the same animals where dLAN treatment promoted weight gain (Figure 1). A total of 1,115,425 16S rRNA gene sequences and 314 amplicon sequence variants (ASVs, Supplementary Table S1) were obtained from all samples. The results of beta diversity by principal coordinates analysis (PCoA) indicated that most of the replicates of each condition tended to be distributed together in space (Figure 2A). In contrast, microbial alpha diversity analysis (Shannon index) showed that samples significantly differed between ZT0 and ZT10 in both LD and dLAN samples (Figure 2B). The taxonomic composition of the bacterial communities in the fecal samples encompassed eight annotated phyla, where Firmicutes was the most abundant in all conditions tested (Figure 2C), decreasing its abundance at ZT10 compared to ZT0 in both LD and dLAN treatments, which is explained by a significant increment of Verrucomicrobia. In addition, the relative abundance (average between ZT0 and ZT10) of Firmicutes was higher in dLAN (87.76%) than in LD (76.47%), while the relative abundance of Bacteroidetes was lower in dLAN (1.08%) than in LD (2.06%), giving an increased Firmicutes over Bacteroidetes ratio in dLAN compared to LD (~2-fold). The changes observed in the taxonomic diversity and composition between the light treatments (LD and dLAN) and between the different sampling times (ZT0 and ZT10) at the same condition highlight the dynamics and plasticity of the mouse colonic microbiome to a non-nutritional condition within a daily cycle.

Given the microbial diversity between ZT0 and ZT10 in both LD and dLAN (Figure 2B), we normalized the abundance of each ASV at ZT0 by its abundance at ZT10 (ZT0/ZT10 ratios) to properly compare the impact of the differential light treatments (LD and dLAN) on the gut microbiome. We standardized the abundance values by ZT10 since, at this time, mice of both groups did not differ in their light treatment (150 lux light for LD and dLAN treated mice) and also because the mice subjected to both light treatments were inactive, there were no other acute effects on the colonic microbiota, such as differences in the feeding pattern of the animals.

**Figure 3:**
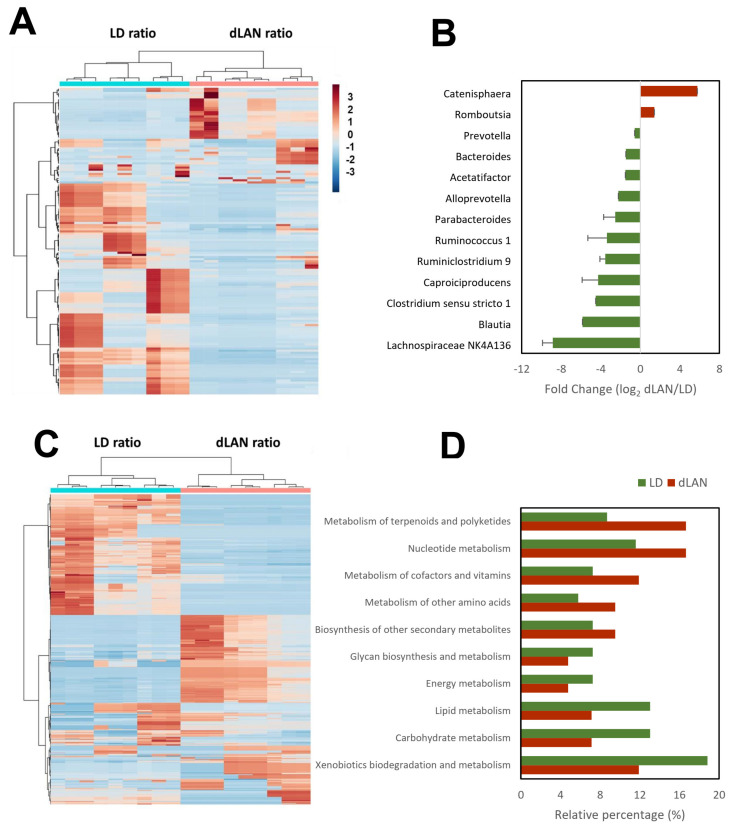
Taxonomic and imputed functional abundances in LD and dLAN at ZT0 and ZT10 conditions. (**A**,**C**) Hierarchical cluster analysis of the bacterial taxonomic and imputed functional compositions, respectively. (**B**) Fold changes (log_2_) of the relative abundances of each genus in dLAN with respect to LD. Over- (red) and under- (green) represented genera in dLAN (*p* < 0.05, Student’s *t*-test; data are shown as means ± SEM). (**D**) Relative percentage of over-represented parent functions of KEGG pathways in LD (green) and dLAN (red) conditions.

The results of the hierarchical cluster analysis indicated that the bacterial communities present in samples from the same light conditions were similarly distributed, perfectly separating LD and dLAN (Figure 3A). Furthermore, we observed that the annotated taxonomic genera Catenisphaera and Romboutsia were more abundant in the dLAN condition, while Lachnospiraceae NK4A136 group, Blautia, Clostridium sensu stricto 1, Caproiciproducens, Ruminiclostridium 9, Ruminococcus 1, Parabacteroides, Alloprevotella, Acetatifactor, Bacteroides, and Prevotella were less abundant in the dLAN condition (over-represented in the LD condition) (Figure 3B). Interestingly, many different ASVs belonged to the Ruminiclostridium 9 and Lachnospiraceae NK4A136 groups (20 and 8, respectively), suggesting that these genera are highly sensitive to light treatments (Supplementary Table S2).

### 2.3. Dim Light at Night Impacts the Biological Pathways in Mouse Colonic Bacterial Communities

To uncover whether the microbial colonic taxonomic composition relates to the overall predicted bacterial functional capacities, we analyzed the imputed biological pathway (KEGG) profiles within the bacterial communities from the different samples by standardizing the functions abundance values by ZT10 (Supplementary Figure S2). Like the clusters formed from the relative abundance ratio of each ASV, the hierarchical cluster analysis of the biological pathways according to EC codes within the bacterial communities formed clusters between samples from the same conditions, separating LD and dLAN (Figure 3C). Regarding the functional responsive processes (Supplementary Table S2), we observed that none of the KEGG parent functions were unique to the microbiomes of mice subjected to each experimental condition. However, we identified that the relative percentage of KEGG pathways: “metabolism of terpenoids and polyketides”, “nucleotide metabolism”, “metabolism of cofactors and vitamins”, “metabolism of other amino acids”, and “biosynthesis of other secondary metabolites” were more abundant in dLAN than in LD, while “glycan biosynthesis and metabolism”, “energy metabolism”, “lipid metabolism”, “carbohydrate metabolism” and “xenobiotics biodegradation and metabolism” were more abundant in LD than in dLAN (Figure 3D).

### 2.4. Dim Light at Night Impacts the Interaction Network of Mouse Colonic Bacterial Communities

**Figure 4:**
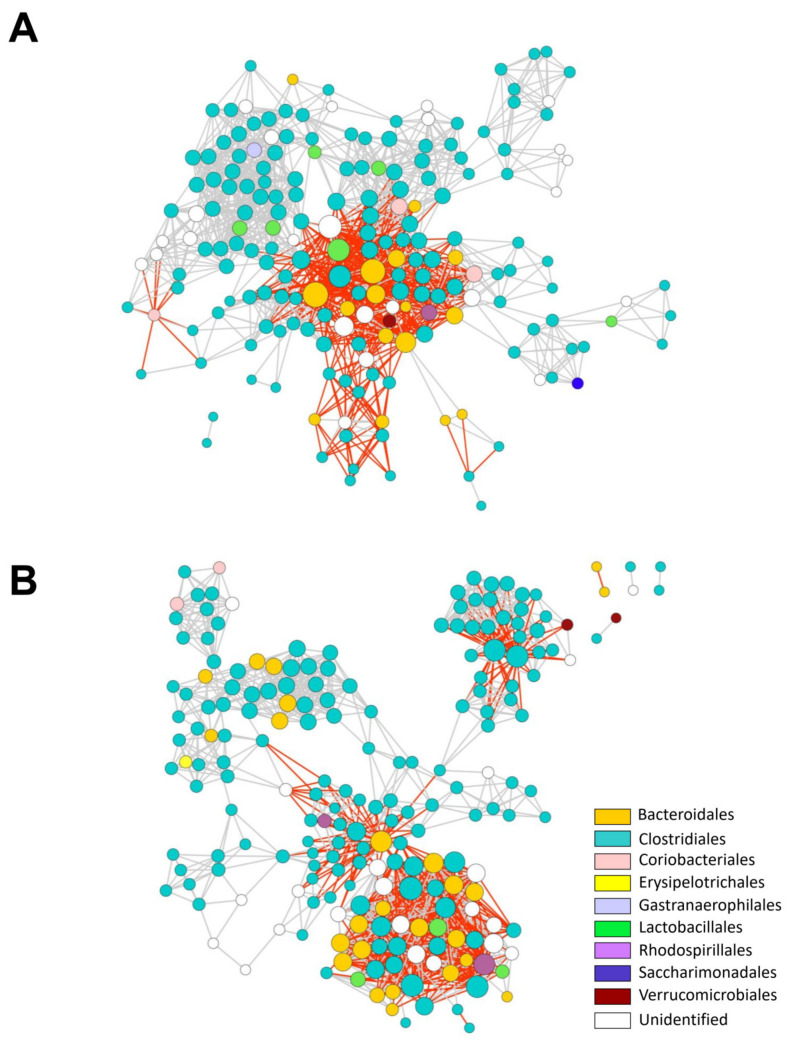
Bacterial interaction networks in mouse colons under LD and dLAN conditions. (**A**) LD bacterial interaction network. (**B**) dLAN bacterial interaction network. Interactions were inferred from ASV abundance patterns. Each node represents an ASV, and each edge represents a significant pairwise association between them (grey lines: positive correlations; red lines: negative correlations). Different node colors represent a distinct order. Node size is proportional to the number of connections (degree) for both networks (maximum node degree for LD is 48 and 37 for dLAN). LD bacterial interaction network contains 1353 connections (887 positive; 466 negative; positive/negative = 1.90); dLAN bacterial interaction network contains 1302 connections (836 positive; 466 negative; positive/negative = 1.79).

To further analyze the microbial communities and the ecological rules guiding community assembly in mice associated with light treatments, we generated microbial interaction networks for LD and dLAN colonic microbial communities (Figure 4). The nodes of the networks represent ASVs annotated at the order level, and edges represent positive (grey) or negative (red) correlations between nodes. The interaction network from LD contained 194 nodes, while the dLAN network was formed by 205 nodes (Figure 4), with 93 ASVs common to both networks, 101 LD exclusive ASVs, and 112 ASVs restricted to dLAN. Most exclusive and shared ASVs from both networks belonged to the Clostridiales order, followed by Bacteroidales, reflecting a conserved microbial diversity between LD and dLAN interacting members (Supplementary Table S3). Moreover, both networks displayed one major connected component that included most nodes and edges and one or four minor connected components (formed by only two nodes) in LD and dLAN networks, respectively (Figure 4). Furthermore, the clustering coefficients (0.653 and 0.705 for LD and dLAN networks, respectively) and the number of connections were similar between networks, with 1353 links in the LD network and 1302 in dLAN. The slightly higher number of interactions in the LD network was also reflected in an increased average number of neighbors (14.08 for LD and 13.18 for dLAN) and network density (0.074 for LD and 0.067 for dLAN) in the network of this bacterial community. Additionally, the LD network presented a higher ratio value of positive over negative links compared to dLAN (1.90 and 1.79, respectively), indicating that the mice subjected to the dLAN treatment contained a colonic microbiota with a lower incidence of positive interactions than the microbiome of LD treated mice (Supplementary Table S3).

Finally, to quantify the variation among the interaction networks, we calculated their total interaction dissimilarity or beta diversity (β_WN_). Since β_WN_ is partitioned into the dissimilarity due to difference in species composition (β_ST_) and dissimilarity due to rewiring of shared species (β_OS_) [19], we could evaluate the contribution of each of these components to the extent of dissimilarity of the networks. Interestingly, the LD and dLAN networks showed β_WN_ values of 0.97, β_ST_ of 0.75, and β_OS_ of 0.22, indicating first that both interaction networks are highly dissimilar, and secondly that the dissimilarity of the network structure has both a compositional and interactive component, with a relative impact of the compositional difference of 77.3% (0.75/0.97) and a relative impact of the interactions of shared species of 22.7% (0.22/0.97). These results reveal that although the networks share similar metrics regarding nodes and edges, their interactions are highly dissimilar, indicating that the different light regimes induce rearrangements on the composition, functions, and interactions of the colonic microbial communities of male mice.

## 3. Discussion

Exposure to artificial light at night causes chronodisruption and is associated with alterations such as weight gain [5,6], disturbance of the daily sleep-wake cycle [20], and altered daily rhythms in nocturnal animals [21].

The association between exposure to dim light at night and weight gain is complex, as it has been shown in rats that no weight gain occurred in response to dim light at night exposure [20]. Moreover, it has been reported that the effects of dim light at night on the daily sleep-wake cycle disturbances are age-dependent [22] and also depend on the duration of the stimulus [23]. In the current study, we did not evaluate the effect of age or the duration of the stimulus on the weight gain, but it is possible that similar relations as those reported for the sleep-wake cycle might occur in our model.

In the present study, weight gain by dLAN was observed in mice (Figure 1C), in agreement with previous studies reporting a similar effect mediated by dLAN in mice, where increased body mass, epididymal fat pad mass, and reduced glucose tolerance were observed in mice subjected to dLAN [5,6]. Moreover, the study by Fonken et al. performed in Swiss Webster mice showed that food consumption does not differ between LD and dLAN treatment when the mice had full access to food (ad libitum), but that an altered feeding pattern was observed in dLAN mice compared to LD mice, where food consumption at the resting phase was higher in dLAN mice [6]. Since the regulation of the composition and function of the gut microbiota is governed mainly by host feeding rhythms [24], we hypothesized that the weight gain observed in mice exposed to dLAN was related to an altered colonic microbiota.

It has been widely described that the intestinal microbiota regulates the host food digestion and absorption, contributes to the maintenance of the epithelial integrity, and produces a broad number of metabolites that impact host physiology [1]. Additionally, alteration of the gut microbiota is also associated with pathological changes in the host, such as weight gain, obesity, and metabolic syndrome [25].

The gut microbiota activity also displays an oscillatory behavior, causing the host intestinal cells to be confronted with the daily variations of microbial species and their metabolites [26,27]. Daily differences in gut microbial composition between ZT0 (8:00 a.m.) and ZT10 (6:00 p.m.) in mice exposed to both LD and dLAN were compared, showing significant differences in the microbial diversity between zeitgeber times (Figure 2B). Interestingly, changes in microbial diversity displayed an inversed pattern between zeitgeber times for LD and dLAN, indicating that light regimes are relevant to daily differences in the colonic microbiota.

Taxonomic analysis indicated that at the phylum level, Firmicutes and Proteobacteria abundances were higher in dLAN than in LD, while Bacteroidetes abundance was lower, showing an increased ratio of Firmicutes to Bacteroidetes in dLAN (Figure 2C). Thus, our results of weight gain in mice subjected to dLAN agree with a plethora of literature showing that obesity and metabolic syndrome are associated with an increase in the Firmicutes to Bacteroidetes ratio and an increase in the relative abundance of Proteobacteria [1,28]. In addition, at the genera level, our results indicated that *Romboutsia* and *Catenisphaera* were more abundant in the dLAN condition. Notably, *Romboutsia* spp. have been previously associated with obesity in rats, mice, and humans [29,30,31] and seem to be prevalent pathobionts in obese mammalians. Moreover, *Romboutsia* species, included as an additive in the diet of mice, showed impaired glucose tolerance and fasting insulin compared with mice fed with standard diet [31], both parameters associated with increments in body weight [5]. Conversely, we found genera under-represented in dLAN, such as *Lachnospiraceae NK4A136 group*, *Blautia*, *Clostridium sensu stricto 1*, *Caproiciproducens*, *Ruminiclostridium 9*, *Ruminococcus 1*, *Parabacteroides*, *Alloprevotella*, *Acetatifactor*, *Bacteroides,* and *Prevotella* (Figure 3B). The *Lachnospiraceae NK4A136 group* and *Blautia* genera are recognized SCFA-producing microbes. Recent evidence shows that SCFAs directly or indirectly regulate processes related to obesity, influencing energy, glucose, and lipid homeostasis control via their metabolites [32]. A recent study in obese mice reported that the abundance of *Lachnospiraceae NK4A136* group and *Blautia* genera increased concomitantly with the concentration of acetate, propionate, and butyrate in response to an oral dose of *Bacteroides* species and a prebiotic [33], curbing weight gain and adiposity in the mice. Remarkably, the *Bacteroides* genus was also under-represented in dLAN, and since *Romboutsia* has been negatively correlated with the levels of acetate and butyrate [34], we suggest that the production and beneficial effects of microbial SCFAs could be impaired in mice subjected to the dLAN treatment. Other less abundant genera in mice subjected to dLAN, such as *Parabacteroides* and *Alloprevotella*, have also been negatively correlated with obesity in mice [35,36].

In addition to the grouping of the profiles obtained from the taxonomic analysis (Figure 3A), the results of the hierarchical cluster analysis indicated that the microbial pathways grouped between samples from the same conditions, perfectly separating LD and dLAN (Figure 3C), which emphasizes the relevance of increased light at night on the structure and functions of the mice colonic microbial communities in mice. Among these metabolic pathways, the relative abundance of “energy metabolism”, “lipid metabolism”, and “carbohydrate metabolism” were higher in LD than in dLAN (Figure 3D). Since these processes appear to be negatively affected by the nocturnal light treatment, the idea that the mechanisms explaining weight gain in mice subjected to dLAN are dependent on these microbial pathways is reinforced (Supplementary Table S3).

Finally, to get a comprehensive view of this comparative analysis between LD and dLAN conditions, we also compared using the Student’s *t*-test (*p* < 0.05) the absolute abundance values of all sampling points of the taxa and functions between both light conditions (i.e., a non-standardized ZT0/ZT10 analysis). As expected, given the microbial community variation during the day in LD and dLAN, we obtained a rather uninformative analysis, where only *Parabacteroides* and *Ruminiclostridium 9* were under-represented in dLAN. In the same context, only the metabolism of xenobiotics by cytochrome P450 (ko00980) KEGG function remained significantly different between the light treatments. This function was under-represented in dLAN compared to LD, as was also observed in the standardized ZT0/ZT10 analysis. Hence, an unbiased and more informative analysis of our data was achieved using a standardized method, given the noted variation in the bacterial colonic community of mice between daytime points (ZT0 and ZT10).

Regarding the microbial networks, even though they were highly alike in their overall network parameters (number of nodes, edges, clustering coefficient, and average number of neighbors), their β_WN_ values showed a strong interaction dissimilarity between LD and dLAN colonic bacterial communities. This dissimilarity was explained mainly by species turnover (β_ST_; 77.3%) and, to a lesser extent, to shared ASVs that interacted differentially in each network (β_OS;_ 22.3%). Thus, the interactions of the exclusive ASVs from each network had the highest impact on the dissimilarity of interactions observed between both networks. Nevertheless, since exclusive network ASVs mainly belong to the same taxonomic levels (mainly Clostridiales and Bacteroidales orders), a possible functional redundancy of the main component that triggers the overall variance of LD and dLAN network structures is implied.

On the other hand, the dLAN network presented a lower ratio value of positive links over negative links compared to LD, suggesting a decreased incidence of mutualistic interactions in response to light-induced chronodisruption [37,38]. Thus, this could be explained by an increased production of antimicrobials (macrolides), by a mechanism dependent on the “metabolism of terpenoids and polyketides” pathway, which was higher in dLAN than in LD (Supplementary Table S2). Interestingly, increased cooperative metabolism has been described to provide health benefits for the host mainly due to competition or negative interactions between ASVs, which are associated with antimicrobial warfare, resource overlap, predation, among others [39,40,41,42], and have the potential to diminish the abundance of cooperative metabolism that may benefit the host [43,44,45,46]. Thus, nocturnal light pollution induced by dLAN resulted in a less efficient community and a less advantageous bacterial community for the host.

It is tempting to speculate about the translational consequences of our findings. However, there are many important variables that were not assessed in the current study, such as the effects of extension time of light exposure or the age of the animals on the weight gain. Finally, the mouse model used here is a nocturnal animal where the bulk of its energy intake occurs during the dark phase of the photoperiod [47]. Therefore, future studies are needed to determine if the mouse can be used as an animal model for studying the association between artificial light exposure at night and overweight in humans.

Finally, a correlation between weight gain and altered colonic microbiota in response to dim light under night treatment was found in the mouse model. This change in the microbiota was not only restricted to the structure, but also to the function and interactions between the microorganisms.

## 4. Materials and Methods

### 4.1. Animals

Animal handling and care followed the Guide for the Care and Use of Laboratory Animals of the Institute for Laboratory Animal Research of the National Research Council. The protocols were approved by the Bioethics Commission of the Universidad Austral de Chile (Protocol 328/218). Eighteen mice between 21 and 65 days old (P21–P65) were used in this study.

C57BL/6J mice (Centro de Estudios Científicos, originally obtained from the Jackson Laboratory) were housed in our animal facility at the Universidad Austral de Chile. All animals used in this study had unrestricted access to water and food (ad libitum). Animals were fed with a standard diet (Prolab RMH 3000, Labdiet). The light sources used in this study were white LED and were calibrated using a lux meter (HI 95500, Hanna) to 150 lux for LD and 5 lux for dLAN, respectively (spectra at 150 and 5 lux shown in Supplementary Figure S3).

### 4.2. Body Mass Measurements

Weaned male mice (P21–P65, *n* = 6 mice for LD and *n* = 6 mice for dLAN) were housed in groups of 3 mice per cage and maintained for two weeks under light/dark (LD) photoperiod (12 h each, lights were turned on at 8:00 a.m.AM and turned off at 8:00 p.m.PM). Two weeks later, mice were allocated to the following two photoperiods: light/dark (LD) (control or standard photoperiod, as described above) or dim light at night (dLAN, consisting of 12 h light (150 lux)/12 h dim light (5 lux) at night) photoperiod and maintained under these conditions for ~30 days (see Figure 1 for details). Mice body mass was measured weekly at the same time.

To determine the effect of dLAN and the treatment extension time on the body mass, we performed a two-way ANOVA followed by SIDAK multiple comparison test analysis using the GraphPad Prism 9 software.

### 4.3. DNA Extraction and Sequencing

Mice from cohort 1 (P65; *n* = 6 for LD and *n* = 6 for dLAN conditions; protocol shown in Figure 1) were anesthetized with isoflurane and euthanized by cervical dislocation. Feces from the proximal colon were extracted and immediately stored at −80 °C in 1.5 mL autoclaved plastic tubes. Mice were euthanized at two time points, ZT0 (8:00 a.m.) and ZT10 (6:00 p.m.). Bacterial DNA was extracted from 200 mg of mouse feces using a QIAamp DNA Stool Mini Kit (Qiagen) according to the manufacturer’s instructions. The DNA integrity was evaluated by electrophoresis in Agilent 2200 TapeStation, and DNA concentration was measured by fluorescence in the Qubit equipment. Then, DNA was stored at 4 °C until use. Microbial DNA was amplified using a bacteria-specific primer set, 28 F (5′-GA GTT TGA TCM TGG CTC AG-3′) and 519 R (5′-GWA TTA CCG CGG CKG CTG-3′), flanking variable regions V1-V3 of the 16 S rRNA gene with barcode on the forward primer [48]. Amplification was performed using the Qiagen Kit HotStarTaq Plus Master Mix under the following conditions: initial denaturation at 94 °C for 3 min followed by 28 cycles, each set at 94 °C for 30 seconds, 53 °C for 40 seconds, and 72 °C for 1 min, with a final elongation step at 72 °C for 5 min. After amplification, PCR products were checked in a 2% agarose gel to determine the amplification’s success and the bands’ relative intensity. PCR products were used to prepare DNA libraries following Illumina TruSeq DNA library preparation protocol. Sequencing was performed at the Molecular Research DNA laboratory (Shallowater, TX, USA) on an Illumina MiSeq platform in an overlapping 2 × 300 bp configuration with a minimum throughput of 20,000 reads per sample.

### 4.4. Sequence Analysis and Taxonomic Identification Using SILVA Database

Microbiome bioinformatics was performed with QIIME 2 2019.10 [49]. Raw sequence data were demultiplexed and quality filtered with the minimal quality median set to 30 using the q2-demux plugin followed by denoising with DADA2 [50] (via q2-dada2). Taxonomy was assigned to amplicon sequence variants (ASVs) using the q2-feature-classifier [51] classify-sklearn naïve Bayes taxonomy classifier against the SILVA_132 97% reference sequences. By doing this, we obtained 1270 ASVs comprising 1,115,425 reads across all samples. For analyses, we selected reads that mapped with ASVs that were identified in at least two out of three replicates to analyze data using representative ASVs from each sample. Using this criterion, 314 ASVs were included in the analyses. The complete set of raw data was deposited in the SRA experiment database PRJNA674401.

### 4.5. Microbial Diversity Analysis

Alpha diversity (Shannon index) was estimated using q2-diversity after samples were rarefied (subsampled without replacement) to 889 sequences per sample. Rarefaction curves for each of these metrics were obtained by serial subsampling in increments of 8000 sequences and ten iterations per increment. Alpha diversity measurements were compared between samples using the ten subsamples of each of the three replicates per sample in an analysis of variance (ANOVA) and Tukey’s post-hoc test. Beta diversity (Bray Curtis dissimilarity) between groups was evaluated by a permutational analysis of multivariate dispersions (PERMDISP) and a permutation-based multivariate analysis of variance (PERMANOVA). Differences between the groups were considered significant if *p* was < 0.05.

### 4.6. Prediction of the Microbial Functional Profiles

MicrobiomeAnalyst server [52] was used to predict the functional microbial profiles of the different colonic microbiomes. The ASV reads abundance table and the ZT0/ZT10 ratio table of the microbiomes were used for the Marker Data Profiling (MDP) stage. Here, three filter steps were applied to eliminate ASVs with reads in only one condition and those ASVs with less than 4 reads in 20% of the samples with low variance. Then, the reads for the remaining ASVs were normalized using the Total Sum Scaling (TSS) procedure. The normalized profiles of reads were used to predict functional profiles using Tax4Fun [53] based in the KEGG Orthology database using the Shotgun Data Profiling (SDP) stage. These normalized profiles were scaled and centered (z-score) and used as input to perform a hierarchical cluster analysis based on Euclidean distance and average linkage between clusters.

### 4.7. Identification of dLAN Markers

To identify the ASVs and the imputed functions (KEGG pathways) associated with the light conditions, the relative abundance of the ZT0/ZT10 ratio of each ASV or function was compared between LD and dLAN conditions. Student’s *t*-test calculated *p*-values, and values <0.01 were acknowledged as significantly different. The data were expressed as the logarithm base 2 of the fold change (log_2_FC) between dLAN over LD; positive values were named over-represented in dLAN, while negative values were named under-represented in dLAN.

### 4.8. Microbial Interaction Networks

To generate the microbial interaction networks, ASV abundances of the complete set of LD or dLAN samples were used. Briefly, significant positive or negative abundance correlations across the samples were identified by the CoNet method [54], which were inferred according to the abundance patterns of pairs of ASVs over the samples using a measure that quantifies the similarity of their distributions. When two ASVs showed a similar abundance pattern over the samples, a positive correlation (co-occurrence) was acknowledged; meanwhile, when they presented an anticorrelation in their abundance pattern, a negative correlation (mutual exclusion) was accepted. After assessing all possible combinations of ASVs in the abundance dataset, all significant pairwise relationships were combined to construct the network [55] using a multiple ensemble correlation. Three similarity measures were calculated: Bray Curtis non-parametric dissimilarity index; Pearson and Spearman rank correlations. For each measure and edge, 1000 renormalized permutation and bootstrap scores were generated according to the work of Faust and Raes [56]. The interaction network model was displayed by Cytoscape [57].

All measures of network beta diversity were calculated using the framework proposed by Poisot et al. [19] using the function betalinkr from the R package bipartite (initially implemented in the betalink package). Following Poisot, total interaction beta diversity or dissimilarity of a pair of networks (β_WN_) was partitioned into the dissimilarity due to difference in species composition (β_ST_) and dissimilarity due to rewiring or of shared species (β_OS_). We used the recommended method for additive partitioning known as commondenom [58].

## Data Availability

The complete set of raw data was deposited in the SRA experiment database PRJNA674401.

## Supplementary Materials

The following are available online at https://www.mdpi.com/article/10.3390/ijms23031673/s1.
