# Supplementary material for: Nocturnal Light Pollution Induces Weight Gain in Mice and Reshapes the Structure, Functions, and Interactions of Their Colonic Microbiota
Source: Int J Mol Sci. 2022 Jan 31;23(3):1673. doi: 10.3390/ijms23031673 (PMC8836271; doi:10.3390/ijms23031673)
Supplement: Supplementary file 1 [file ijms-23-01673-s001.zip › Supplementary Figure S1.pdf]

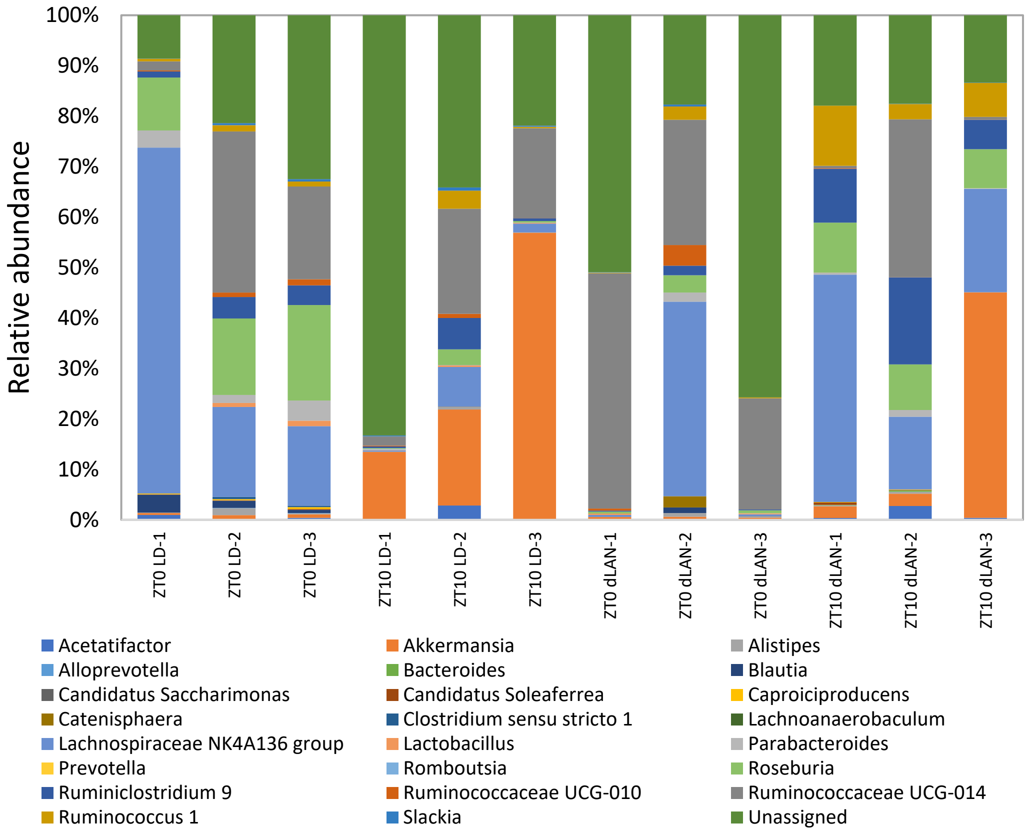

Supplementary Figure S1. Taxonomic structure of bacterial communities from LD and dLAN samples at genus level. Average bacterial genera relative abundance in all samples.

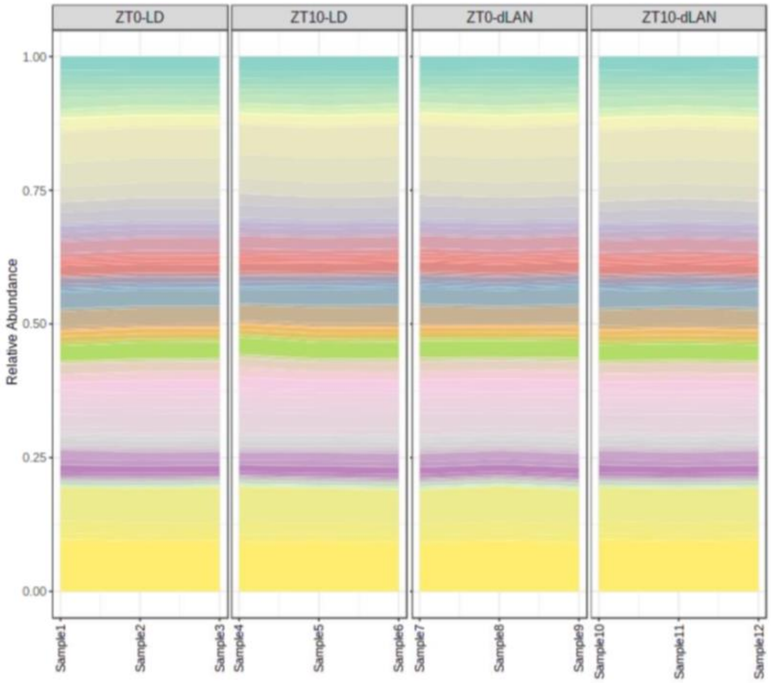

Supplementary Figure 2. Imputed functional capacities of the colonic bacterial communities. Relative abundance profiles the imputed functional capacities of the bacterial communities from LD and dLAN samples at ZT0 and ZT10. Different colors represent different functions

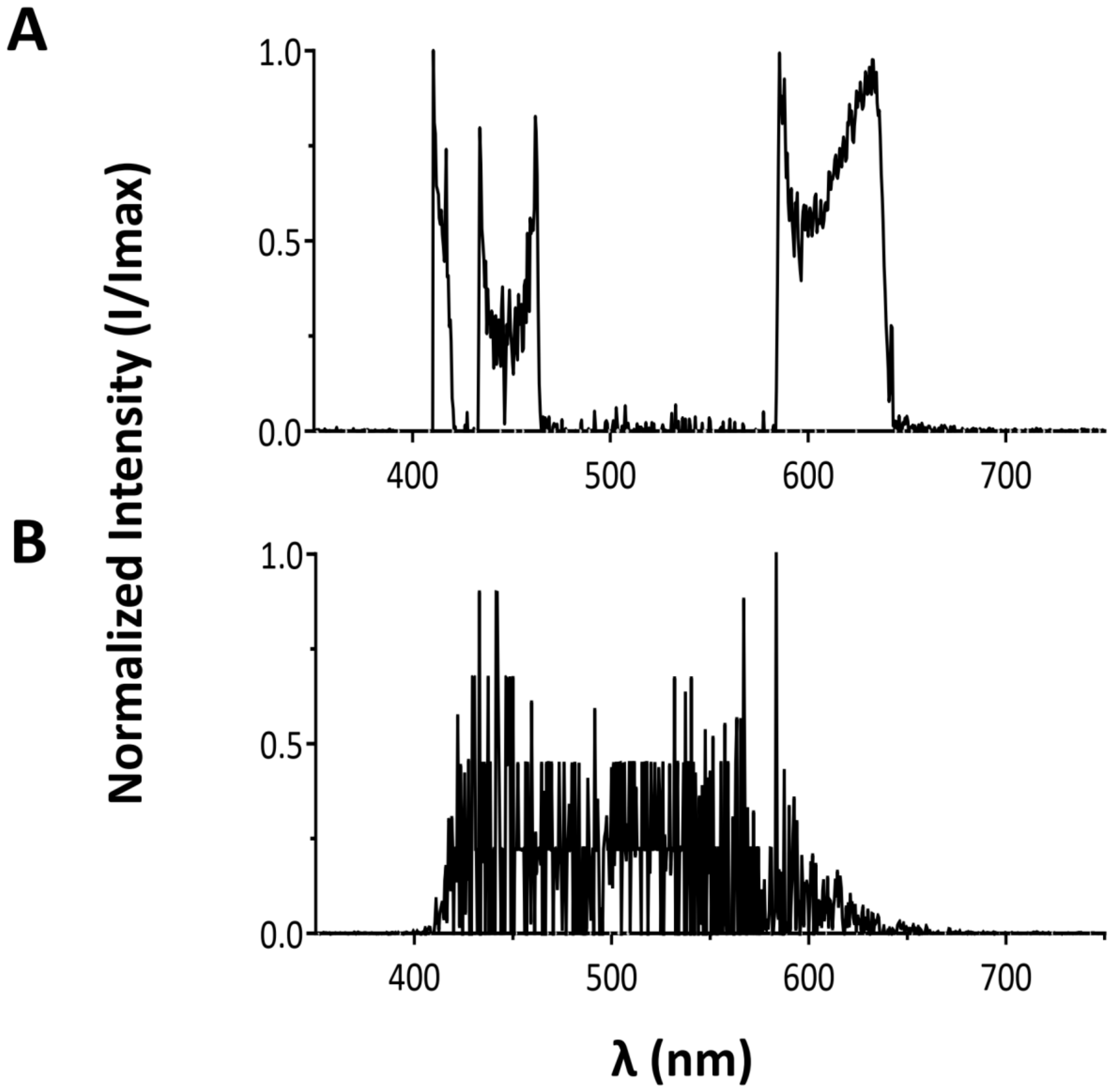

Supplementary Figure 3. Emission spectra from light emitting diodes (LED). A. The normalized emission spectrum from a white LED adjusted to obtain 150 lux of illuminance. B. normalized emission spectra, from the same LED in A, but adjusted to obtain 5 lux of illuminance.
